# CRISPR/Cas9-mediated heterozygous knockout of the autism gene *CHD8* and characterization of its transcriptional networks in neurodevelopment

**DOI:** 10.1186/s13229-015-0048-6

**Published:** 2015-10-19

**Authors:** Ping Wang, Mingyan Lin, Erika Pedrosa, Anastasia Hrabovsky, Zheng Zhang, Wenjun Guo, Herbert M. Lachman, Deyou Zheng

**Affiliations:** Department of Neurology, Albert Einstein College of Medicine, 1300 Morris Park Ave, Bronx, NY 10461 USA; Department of Genetics, Albert Einstein College of Medicine, 1300 Morris Park Ave, Bronx, NY 10461 USA; Department of Psychiatry and Behavioral Sciences, Albert Einstein College of Medicine, 1300 Morris Park Ave, Bronx, NY 10461 USA; Department of Cell Biology, Albert Einstein College of Medicine, 1300 Morris Park Ave, Bronx, NY 10461 USA; Gottesman Institute for Stem Cell and Regenerative Medicine Research, Albert Einstein College of Medicine, 1300 Morris Park Ave, Bronx, NY 10461 USA; Department of Neuroscience, Albert Einstein College of Medicine, 1300 Morris Park Ave, Bronx, NY 10461 USA; Department of Medicine, Albert Einstein College of Medicine, 1300 Morris Park Ave, Bronx, New York USA

**Keywords:** Autism, ASD, *CHD8*, CRISPR/Cas9, Induced pluripotent stem cells, iPSC, Neurodevelopment, Macrocephaly, RNA-seq, Schizophrenia

## Abstract

**Background:**

Disruptive mutation in the *CHD8* gene is one of the top genetic risk factors in autism spectrum disorders (ASDs). Previous analyses of genome-wide *CHD8* occupancy and reduced expression of *CHD8* by shRNA knockdown in committed neural cells showed that *CHD8* regulates multiple cell processes critical for neural functions, and its targets are enriched with ASD-associated genes.

**Methods:**

To further understand the molecular links between *CHD8* functions and ASD, we have applied the CRISPR/Cas9 technology to knockout one copy of *CHD8* in induced pluripotent stem cells (iPSCs) to better mimic the loss-of-function status that would exist in the developing human embryo prior to neuronal differentiation. We then carried out transcriptomic and bioinformatic analyses of neural progenitors and neurons derived from the *CHD8* mutant iPSCs.

**Results:**

Transcriptome profiling revealed that *CHD8* hemizygosity (*CHD8*^+/−^) affected the expression of several thousands of genes in neural progenitors and early differentiating neurons. The differentially expressed genes were enriched for functions of neural development, β-catenin/Wnt signaling, extracellular matrix, and skeletal system development. They also exhibited significant overlap with genes previously associated with autism and schizophrenia, as well as the downstream transcriptional targets of multiple genes implicated in autism. Providing important insight into how *CHD8* mutations might give rise to macrocephaly, we found that seven of the twelve genes associated with human brain volume or head size by genome-wide association studies (e.g., *HGMA2*) were dysregulated in *CHD8*^+/−^ neural progenitors or neurons.

**Conclusions:**

We have established a renewable source of *CHD8*^+/−^ iPSC lines that would be valuable for investigating the molecular and cellular functions of *CHD8*. Transcriptomic profiling showed that *CHD8* regulates multiple genes implicated in ASD pathogenesis and genes associated with brain volume.

**Electronic supplementary material:**

The online version of this article (doi:10.1186/s13229-015-0048-6) contains supplementary material, which is available to authorized users.

## Background

ASDs are a class of neurodevelopmental disorders characterized by persistent deficits in social communication/interaction and restricted, repetitive patterns of behaviors, interests, or activities (DSM-5) [1]. The genetic risk factors for ASD are heterogeneous, and up to 1 thousand genes are estimated to be involved [2]. Recent whole exome and genome-sequencing projects, focused on the identification of rare de novo mutation in the probands of ASD family “trios” or “quads”, have discovered hundreds of genes with functionally disruptive mutations [3–12], some of which also map to ASD-associated de novo copy number variations (CNVs) [13]. Many of the implicated genes encode proteins involved in synapse formation, transcriptional regulation, and chromatin remodeling [10, 14], indicating a likely convergence of functional pathways, despite genetic heterogeneity.

Chromodomain helicase DNA binding protein 8 (*CHD8*) emerged as a top ASD-candidate gene from multiple exome-sequencing studies [6, 8], which altogether have analyzed thousands of ASD probands and in some cases their families too [15, 16]. More importantly, disrupting *chd8* in zebrafish development has recapitulated multiple features of ASD, including macrocephaly observed in some ASD cases carrying *CHD8* mutations [15, 16]. In addition, macrocephaly is often found in ASD caused by disruption of other candidate genes [17].

*CHD8* is a ubiquitously expressed member of the CHD family of ATP-dependent chromatin-remodeling factors that play important roles in chromatin dynamics, transcription, and cell survival [18]. Previous studies [19–21] have shown that the *CHD8* protein interacts with β-catenin and negatively regulates Wnt signaling, while both β-catenin and Wnt signaling play critical roles in normal brain development and neuropsychiatric disorders [22], including ASD [14]. *CHD8* also functionally interacts with p53 [23] and recruits MLL histone methyltransferase complexes to regulate cell cycle genes [24].

To elucidate the roles of *CHD8* in neurodevelopment and address the contribution of its disruption to ASD pathogenesis, three groups have recently reported genome-wide *CHD8* binding sites and transcriptomic changes upon shRNA-mediated knockdown of *CHD8* expression in neural progenitor cells (NPCs) [25], neural stem cells (NSCs) [26], and SK-N-SH neuroblastoma cells [27]. The results showed that *CHD8* binds to thousands of genes, largely biased to promoters, in NPCs, NSCs, and developing mammalian brains. Reduced expression of *CHD8* resulted in the potential disruption of gene networks involved in neurodevelopment, which contained many ASD-risk genes [15, 25–27]. The transcriptional targets of *CHD8* have also been studied in non-neural systems, as it is also involved in cell cycle regulation, Wnt signaling, and several forms of cancers [19, 21, 24, 28, 29].

As ASD is a developmental disorder with symptoms emerging in early childhood and the *CHD8* causal mutations in patients are germline, it is important to establish cell models that can mimic the persistent loss of *CHD8* function in the developing embryos prior to and during neuronal differentiation and brain development. Therefore, we applied CRISPR/Cas9 technology [30] to generate *CHD8*^*+/−*^ iPSCs by knocking out one copy of the gene in an iPSC line derived from a healthy male subject. We then differentiated both the wild-type (WT) and the *CHD8*^*+/−*^ iPSCs to NPCs and subsequently neurons and performed comparative transcriptomic analysis (RNA-seq). Our approach has several advantages: precisely targeted changes at the DNA level, persistent reduction of *CHD8* expression, no introduction of extra genetic materials (e.g., virus vector) to the cells, and greater flexibility in the types of differentiated cells that can be generated.

We found that heterozygous *CHD8* knockout (KO) disrupted the expression of many genes involved in extracellular matrix formation, neuronal differentiation, and skeletal system development. Interestingly, *CHD8*-regulated genes were enriched with ASD-risk genes, schizophrenia-risk genes, and genes implicated in regulating head size or brain volume. Furthermore, we found that *CHD8*-regulated genes significantly overlapped with the downstream targets of several critical genes (*TCF4*, *EHMT1*, *SATB2*, and *NRXN1*) that have been associated with ASD or other neuropsychiatric disorders. Taken together, our results not only shed light on the molecular roles of *CHD8* in neurodevelopment, but also provide evidence of potential convergence of cellular pathways that could be disrupted by mutations of distinct ASD-risk genes.

## Methods

### Development of iPSCs from skin fibroblasts

We have been developing iPSCs from controls and patients with 22q11.2 deletion and diagnosis of a psychotic disorder [31]. The study and consent forms were approved by the Institutional Review Board of Albert Einstein College of Medicine . Consent was obtained by a skilled member of the research team who had received prior human subject training. One of the healthy male control (without 22q11.2 del too) was used in the current study. Exome sequencing was performed on DNA extracted from white blood cells of this subject to detect coding variants prior to generating the *CHD8* KO. We used GATK [32] for variant calling and ANNOVAR [33] for variant annotation.

The iPSC line used in this study was generated from fibroblasts obtained from a skin biopsy performed by a board-certified dermatologist. The procedures for growing fibroblasts and iPSC reprogramming are detailed in Additional file 1. Pluripotency was confirmed by immunocytochemistry using antibodies (Ab) against Tra-1-60, Tra-1-81, SSEA3, and SSEA4, which are expressed in pluripotent stem cells. In addition, the capacity to differentiate into all three germ layers was established by in vitro assays, as previously described (see Additional file 1 for details) [34, 35].

### Design of the *CHD8* single guide RNA sequences

Single guide RNA (sgRNA) sequences targeting the region adjacent to the Ser62 codon of *CHD8* were picked using the online CRISPR design tool [36] from the Zhang lab at the Broad Institute, and the two selected sgRNAs were predicted to have very low probability off-target sites. The sgRNA sequences were then cloned into the pSpCas9 (BB)-2A-Puro (PX459) vector (a gift from Dr. Feng Zhang, Addgene plasmid # 48139) [30].

### CRISPR/Cas9-mediated *CHD8* knockout

Human iPSCs were cultured and fed daily in mTeSR1 (Stem Cell technologies) on Matrigel (BD)-coated plates at 37 °C/5 % CO_2_/85 % in a humidified incubator. Cells were maintained in log phase growth, and differentiated cells were manually removed. iPSCs were exposed to 10-μM ROCK Inhibitor for ~4 h to improve cell survival during nucleofection. After 4 h, growth medium was aspirated, and the cells were rinsed with DMEM/F12. iPSCs were dissociated into single cells using accutase and harvested. Nucleofection was performed using the Amaxa-4D Nucleofector Basic Protocol for Human Stem Cells (Lonza) according to the manufacturer’s instructions. Briefly, 8 × 10^5^ cells and 5 μg of the CRISPR/Cas9 plasmids with either sgRNA1 or sgRNA2 were nucleofected using the P3 Primary Cell 4D-Nucleofector X Kit L with program CA-137. Cells were resuspended in mTeSR1 + 10-μM ROCK Inhibitor and placed in one well of a 6-well Matrigel-coated plate. The following day, cells were fed with fresh mTeSR1, and were subsequently fed with fresh medium every day. On days 4–14, cells were exposed to 0.5 μg/ml puromycin for 6 h. Puromycin-resistant colonies were picked and expanded in mTeSR1 without further puromycin treatment.

### Characterizing *CHD8* knockout lines

“TA” cloning was used to identify the knockout alleles. A 479-bp PCR amplicon flanking the CRSPR/Cas9-targeted sites was generated using the primers 5’-CTGTAAGACAGGTTGGGCTG-3’ and 5’-CTTGTTTCTTGCCTCTATACTTGA-3’. The PCR product was purified and ligated into pCR™2.1 using a TA Cloning Kit developed by Life Technologies following the manufacturer’s protocol. Recombinant plasmids were introduced into competent *E. coli* and selected in ampicillin. Plasmid DNA was extracted and sequenced across the insert using one of the PCR primers.

Western blotting confirmed that the *CHD8*^*+/−*^ lines expressed lower levels of *CHD8* protein. Specifically, cell lysates from NPCs differentiated from WT, and KO iPSCs were separated by SDS PAGE, transferred to PVDF membranes, and then blotted with anti-*CHD8* antibody (Bethyl Cat #A301-224A). Anti-actin antibody (BD Biosciences, Cat # 612656) was used for loading control.

### Neuronal differentiation

Neurons were generated from iPSC-derived NPCs as described by Marchetto et al. with slight modifications [34, 37]. A detailed description of the protocol can be found in Additional file 1. Essentially, the protocol leads to a mixed population of glutamatergic and GABAergic neurons, from which RNA was extracted and sent for sequencing.

### RNA-seq analysis

We obtained 101-bp paired-end RNA-seq reads from Illumina HiSeq 2500. RNA-seq reads were aligned to the human genome (hg19) using Tophat (version 2.0.8) [38]. The number of RNA-seq fragments mapped to each gene was determined for genes in the GENCODE database (v18) [39]. Exonic/intronic/intergenic rates were calculated by CollectRnaSeqMetrics in Picard [40]. Cufflink (version 2.2.1) [41] was used to generate the gene-expression values as FPKMs (fragments per kilobase of exon per million fragments mapped). We restricted our analysis to 12,843 protein-coding genes with average FPKM >1 across all four samples. DESeq2 [42] was used to determine differentially expressed genes (DEGs) in NPCs and neurons. The list of significantly DEGs was defined at false discovery rate (FDR) < 0.05. DAVID [43, 44] was used for Gene Ontology (GO) analysis with 12,843 expressed protein-coding genes as background. Ingenuity pathway analysis (IPA) [45] was used for canonical pathway analysis and disease association, with the ingenuity knowledge base (genes only) as background. Toppgene [46] was used for human phenotype ontology analysis, and the whole genome was used as background. The RNA-seq data have been deposited in the Gene Expression Omnibus (GEO; accession # GSE71594).

To find neurodevelopment genes specifically affected by *CHD8*^*+/−*^, we added an interaction design in DESeq2 (option: ~celltype + genotype + celltype:genotype) to specifically model the interactions between development status (NPCs or neurons) and *CHD8* status (WT or KO).

### Validating targeted deletions and assessing off-targets using RNA-seq reads

First, we examined if *CHD8* was targeted and edited precisely according to our CRISPR sgRNA design. A 2-bp deletion in chr14:21899785 (hg19) and a 10-bp deletion in chr14:21899722 (hg19) were identified in a proportion of RNA-seq reads that mapped to targeted regions of the two CRISPR sgRNAs in *CHD8*^*+/−*^ samples (KO1 and KO2, respectively). This was confirmed by DNA sequencing. The two short indels were not found in any of the WT samples. We also called short indels (supported by at least five RNA-seq reads) from RNA-seq reads by samtools [47], but we did not detect any additional indels that were present in *CHD8*^*+/−*^ samples but not in the WT controls.

### Quantitative real-time PCR (qPCR)

qPCR was carried out on reverse transcribed PCR using the 2^−ΔΔCt^ method as previously described [48, 49]. A detailed description and the primers used for this analysis can be found in Additional file 1.

### Definition of *CHD8-*binding genes

ChIP-seq peaks of *CHD8*-binding sites in NPCs were from Sugathan’s report [25]. Only peaks replicated by all three antibodies were used. Genes with at least one peak from 2 kb upstream of the transcription start sites to the transcription terminus were defined as *CHD8*-binding genes.

### Interaction network analysis

DEGs in NPCs with *CHD8*-binding were imported into the STRING database v10 [50] to construct protein-protein interaction networks. We retained any interaction (i.e., edge) from experiments and databases that had a median confidence ≥0.4.

For detecting converged networks of multiple ASD-risk genes, we first collected DEG lists from our *CHD8*^*+/−*^ NPCs, *TCF4* knockdown, *EHMT1* knockdown, *MBD5* knockdown, and *SATB2* knockdown, respectively [51, 52], and then imported genes shared by at least two lists into the STRING database to construct gene networks. GO enrichment was calculated by “Enrichment” function in STRING. Networks were visualized using Cytoscape [53]. The same approach was also applied to DEGs from *CHD8* KO, *ZNF804A* KD, and *NRXN1* KD neurons.

### Upstream regulator prediction

IPA was used to predict upstream regulators for 841 DEGs without *CHD8* binding in NPCs. In this analysis, the *p* value from IPA measures the significance in overlap between query genes and pre-defined sets of genes that are regulated by a specific regulator, using the Fisher test. At the end, we used *p* < 0.05 to select upstream regulators that (a) regulated at least five non *CHD8*-bound DEGs and, themselves, were (b) in our NPC DEG list.

### ASD/schizophrenia-risk gene sets

The first ASD gene set was obtained from the SFARI gene-scoring module [54], using genes scored as high confidence, to minimal evidence and syndromic. The second ASD gene set was from the AutismKB [55] core dataset, which includes syndromic autism related genes and non-syndromic-related genes, designated as high confidence. High-confidence and probable ASD genes in Willsey’s paper [56] were used as the third set (“Willsey_ASD”). Genes predicted from whole exome-sequencing and co-expression network [57] were used as another set (“Liu_ASD”). The other two lists were derived from massive whole exome sequencing: one (“Iossifov_ASD”) focused on de novo mutations [11] and the other (“DeRubeis_ASD”) combined de novo and inherited mutations to develop a high-confidence list (FDR < 0.1) [10]. Two schizophrenia gene lists were from the SZgene database [58] and a recent GWAS report [59].

### Identification of common GO terms for DEG lists from different *CHD8* studies

DEGs were determined by the following criteria from data in four previous studies. For the study by Cotney et al. [26], we selected genes with logFC > 0.1 and log counts per million (logCPM) between 2 and 10 to meet the Poisson assumption, as described by the original authors. However, we repeated differential expression analysis with a less stringent FDR cutoff, using the Benjamini-Hotchberg method instead of Bonferroni to adjust *p* values for significance. For the study by Sugathan et al. [25], we used ComBat in the sva package [60] to adjust batch effects, followed by differential expression analysis with DESeq2 [42]. DEGs were selected by *p* value < 0.05; 96 % of DEGs in Sugathan’s list were in our reanalysis list. For Wilkinson et al’s study [27], we used the DEG list provided by the authors.

Enriched GO terms for each of the five DEG lists were first determined by ClueGO [61] (*p* value < 0.05). Subsequently, GO terms shared by ≥3 DEG lists were considered as common and used for subsequent analysis of function overlap between the five *CHD8*^*+/−*^ and knockdown studies. The relationships among the selected GO terms were based on their shared genes, which was measured using kappa statistics [62]. Two GO terms were connected by an edge if they had a kappa score >0.4. ClueGO relies on term similarity to define functional groups of multiple terms. In our analysis, we set initial group size to 5 (default value, 2) and percentage for group merge (default values, 50 %) to 80 % to obtain a summary of less redundant functional clusters of common GO terms. Since a GO term can be included in several functional groups, we assigned each term only to the functional groups in which it had the most significant group *p* values, meaning that this term had the most similar genetic component in this group. Subsequently, terms of the same groups formed a closed circle. In addition to edges connecting terms to show their relationships, we also added edges between terms and studies to reveal enrichment of specific GO terms among individual DEG lists.

### Statistics

To test if DEGs were significantly overlapped/enriched with a specific gene set, 12,843 expressed genes in our samples were used as background (of all genes) for the Fisher exact test. Statistics tests were conducted in R [63] and multiple test correction was applied unless specified otherwise.

## Results

### Characterization of *CHD8* knockout iPSC lines

**Fig. 1:**
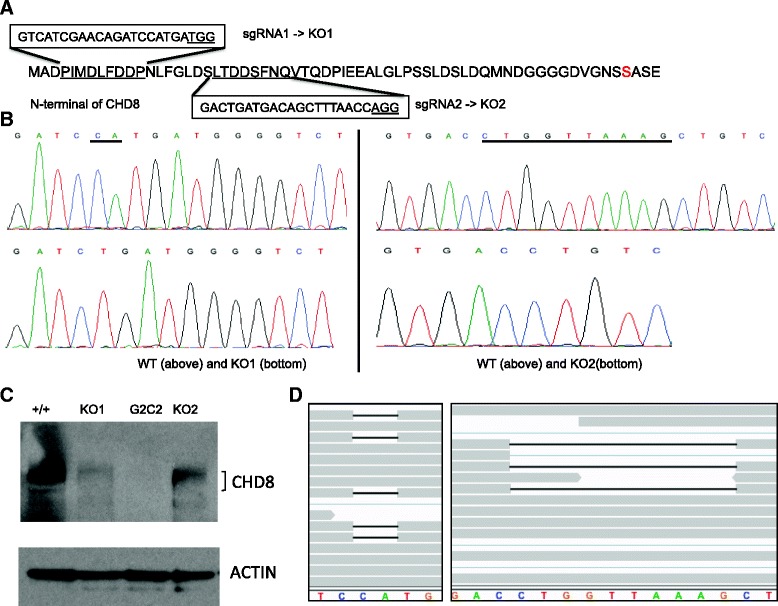
Generation and characterization of *CHD8* ^*+/−*^ lines. **a** Design of CRISPR guide sgRNA sequences targeting the N-terminal end of *CHD8*. The *red marks* Ser62 where premature stop codon mutations were uncovered from whole exome-sequencing analysis of ASD individuals [16]. **b** DNA-sequencing analysis of *CHD8* ^*+/−*^ iPSC clones. Knockout alleles were identified by cloning of PCR products and Sanger sequencing. **c** Western blot analysis of *CHD8* ^*+/−*^ NPCs. *CHD8*-specific antibodies were used to detect *CHD8* protein in NPC lysates. A clone with homozygous *CHD8* knockout (G2C2) was also analyzed, but this clone could not differentiate into neurons appropriately and thus was not included in our RNA-seq analysis. **d** Validation of 2-bp (KO1, *left*) and 10-bp (KO2, *right*) deletion by RNA-seq reads. A screen shot of RNA-seq reads mapped to the CRISPR targeting regions, with gap showing deletion

Several functional disruptive mutations, including premature stop codons and frameshift mutations, have been detected in *CHD8* in ASD probands [15, 16]. We designed two separate CRISPR sgRNA sequences to target the N-terminal of *CHD8* protein to generate truncated mutations (Fig. 1a). iPSCs derived from a healthy male subject were transduced with CRISPR/Cas9 vectors containing each of the two target sequences. After screening, two clones, one with a 2-bp (KO1) and the other with a 10-bp (KO2) heterozygous deletion were found; the other allele was intact in both (Fig. 1b). Both genomic DNA sequencing and Western blotting analysis (Fig. 1b, c) confirmed the heterozygous knockout status for both clones. The *CHD8*^*+/−*^ iPSC lines were used to generate NPCs and early differentiating neurons for RNA-seq analysis, together with samples prepared from the parental WT clones, for a total of eight samples (two biological replicates of WT and *CHD8*^*+/−*^ at two neurodevelopmental stages).

### Genes with altered expression in *CHD8*^*+/−*^ are involved in neurodevelopment

During RNA-seq analysis, we obtained on average ~28 million read pairs per sample (Additional file 2: Table S1). Examination of the RNA-seq reads mapped to the *CHD8* exons confirmed the 2-bp and 10-bp deletion (Fig. 1d), indicating that both the WT and KO *CHD8* copies were expressed in NPCs and neurons, with fewer reads from the KO copy than the WT. Importantly, our indel analysis of the RNA-seq reads detected no off-target sites at coding regions (see Methods).

**Fig. 2:**
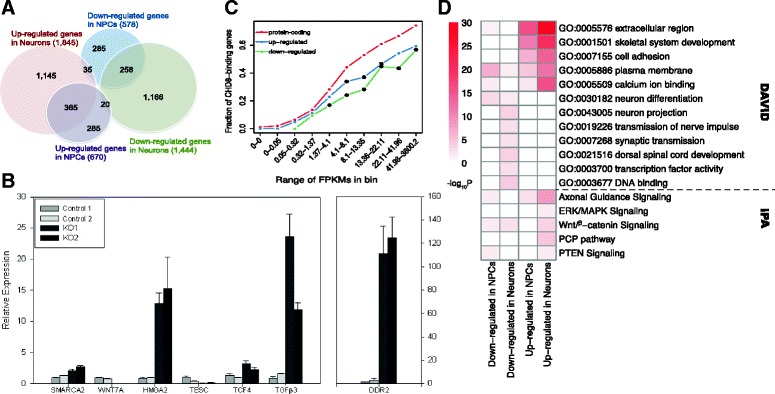
Functional annotation of DEGs and their relationship to *CHD8*-binding. **a** Venn diagram of DEGs from *CHD8* ^*+/−*^ in iPSC-derived NPCs and neurons. **b** qRT-PCR validation of seven DEGs, using β2-microglobulin (β2M) as a reference gene to calculate relative expression levels. The qPCR was carried out on neurons derived from two control samples and the two *CHD8* ^*+/−*^ clones (the same samples used for the RNA-seq). Each was normalized against another control sample. Samples were analyzed in triplicate. The *graph* shows the fold change for each sample relative to the neutral control, *error bars* show the +/− standard deviation. The fold changes were highly significant: *SMARCA2*, *p* = 9.7E-7; *WNT7A*, *p* = 6.1E-9; *HMGA2*, *p* = 4.4E-6; *TESC*, *p* = 0.003; *TCF4*, *p* = 4.4E-5; *TGFB3*, *p* = 5.5E-6; *DDR2*, *p* = 6.2E-8 (two-tailed Student’s *t* test). **c** Percentage of *CHD8*-binding genes in all protein-coding, upregulated, and downregulated genes in NPCs, ranked by their expression values (FPKMs) in WT NPCs. *Asterisks* (“*”) mark the groups of genes showing a significant difference in the proportions of genes with *CHD8* binding by comparing DEGs with all protein-coding genes (binomial test, two-tailed, *p* < 0.05). **d** Representative-enriched GO terms (*top*) and canonical pathways (*bottom*) among DEGs, as reported by DAVID and IPA, respectively. The *red color* in each cell corresponds to the −log_10_(*p value*), corrected by Benjamini-Hotchberg method (*color scale* on the *left* and only terms with *p* < 0.05 were shown)

Differential expression analysis identified 1248 and 3289 genes that changed expression in NPCs and neurons, respectively (FDR < 0.05, Fig. 2a, Additional file 3: Table S2). Note that *CHD8* showed no significant expression change at the transcript level (*p* = 0.85 in NPCs, *p* = 0.27 in neurons, Additional file 3: Table S2). The fact that many more DEGs were detected in neurons than NPCs indicates that persistent *CHD8* hemizygosity could have continuous and amplified effects in neurodevelopment, i.e., genes with altered expression in NPCs would directly affect the expression of additional sets of genes in the differentiating neurons. Seven DEGs, including *SMARCA2*, *WNT7A*, *HMGA2*, *TESC*, *TCF4*, *TGFβ3*, and *DDR2*, which are involved in transcription regulation, cell division, and WNT-β-catenin signal pathways, or related to head size or brain volume (see below), were selected for qRT-PCR analysis, and their differential expression in *CHD8*^*+/−*^ neurons was confirmed (Fig. 2b). Of them, *SMARCA2*, *HMGA2*, and *TCF4* were potentially direct targets as they were bound by *CHD8* [25].

Previous studies have reported that *CHD8* bound to thousands of genomic regions (i.e., peaks), especially to gene promoters in NPCs or NSCs [25, 26]. To explore the relationship between DNA binding and gene regulation, we integrated *CHD8* binding in control NPCs [25] with our expression data. Based on the average gene expression of the two WT NPC samples, we separated genes into 10 bins of equal size (1284 genes per bin). Consistent with the results from Sugathan et al. [25], *CHD8* binding was observed more frequently at more highly expressed genes (Fig. 2c). To our surprise, we found that the percentages of CHD8 binding genes among the DEGs were significantly lower than the expectation from genome-wide binding (Fig. 2c). Nevertheless, this finding is consistent with previous reports that only a small percentage of *CHD8*-bound genes displayed differential expression upon *CHD8* knockdown [25]. This result indicates that *CHD8* directly regulates a limited number of genes and that the majority of gene-expression changes due to *CHD8* knockout are indirectly regulated targets.

GO analysis using DAVID revealed that upregulated genes in *CHD8*^*+/−*^ NPCs and neurons were enriched with similar GO terms, including “extracellular region,” “skeletal system development,” and “cell adhesion.” On the contrary, for downregulated genes, except for the GO term “neuron differentiation” that was enriched in both NPCs and neurons, “neuron projection,” “synaptic transmission,” and “transcription factor activity” were only enriched in neurons (Fig 2d, see full list in Additional file 4: Table S3). In addition, using IPA, we found that DEGs were highly enriched in “axonal guidance signaling,” “WNT-β-catenin signal,” and “PTEN signaling” (Fig. 2d, full list in Additional file 5: Table S4). These results indicate that heterozygous *CHD8* mutations may disrupt multiple processes of neurodevelopment and neural functions in ASD. It should be noted that, in addition to nervous system development, the DEGs were also enriched for cancers, gastrointestinal disease, and cardiovascular system development, based on the IPA analysis (Additional file 5: Table S4).

As genes downregulated in both *CHD8*^*+/−*^ NPCs and neurons were enriched for neuronal differentiation, we wondered how heterozygous *CHD8* KO might affect gene regulation during the transition from NPCs to differentiating neurons. To address this, we utilized a two-factor linear model implemented in DESeq2 to search for genes whose transitional expression from NPCs to neurons were affected by *CHD8* reduction (i.e., interaction between neuronal differentiation and *CHD8* status; see Methods). This analysis identified 1098 genes. Among them, 360 also showed a significant differential expression between NPCs and neurons in WT but not in the comparison of *CHD8*^*+/−*^ samples. Of these 360 genes, 207 genes were expressed at a higher level in WT neurons (vs. WT NPCs). GO analysis revealed that these genes were enriched in “neurological system process,” especially in “transmission of nerve impulse” and “synaptic transmission” (Additional file 2: Figure S1A). The 153 genes with higher expression in WT NPCs (vs. WT neurons) were enriched for “cell junction” and “cell adhesion” (Additional file 2: Figure S1B). These results indicate that normal synapse formation and function could be disrupted during *CHD8*^*+/−*^ neuron differentiation.

### *CHD8* direct targets vs. indirect targets

**Fig. 3:**
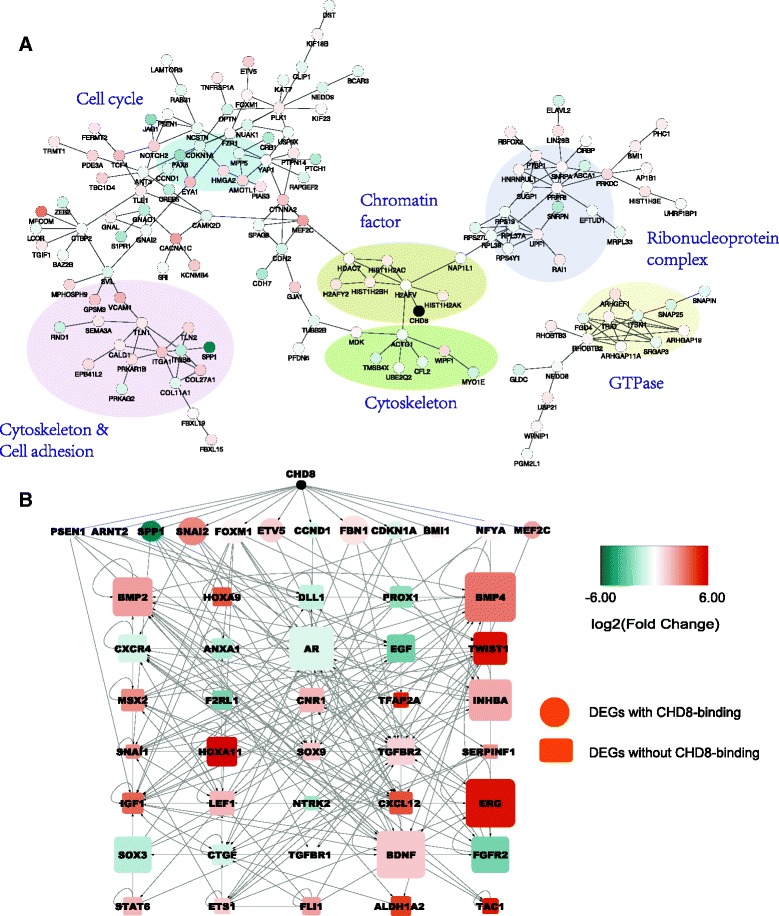
Interaction network of *CHD8* direct targets and a putative *CHD8* regulatory network in NPCs. **a** Functional interaction network generated by the STRING database for *CHD8* direct targets, with *nodes* representing genes and edges representing interactions. *Colors* of the nodes indicate expression changes. Disconnected genes were not shown. **b** A putative regulatory network connecting *CHD8* to a set of upstream regulators, which in turn could regulate the expression of many genes indirectly targeted by *CHD8*. The *edges* represent regulatory relationship predicted by the IPA for upstream regulators that were bound (*ellipse*) or not bound (*rectangle*) by *CHD8. Colors* of the *nodes* indicate expression changes. *Sizes* of the *nodes* show the –log_10_ (*p* value) from IPA. *Arrows* start from an upstream regulator to its targets. Note that only differentially expressed upstream regulators were included in the network

To better understand the regulatory roles of *CHD8*, we further characterized the 407 DEGs in NPCs with *CHD8* binding (i.e., direct targets) and used STRING to define their function interactions, which include direct protein-protein interactions and indirect functional associations. Of the 407 genes, 140 were included in the resulted STRING network, and interestingly, those genes could be grouped into several function clusters: cell cycle, cytoskeleton, cell adhesion, chromatin factor, ribonucleoprotein complex, and GTPase genes (Fig. 3a), revealing major pathways that could be directly regulated by *CHD8* in NPCs. We did not perform the same analysis for neuron DEGs because no *CHD8*-binding data was available for human neurons.

As DEGs were statistically depleted of *CHD8* binding, we wondered how then those 841 NPC DEGs without *CHD8* binding (i.e., indirect targets) could be affected by reduced *CHD8* expression. To address this, we applied IPA software to predict the upstream regulators of these *CHD8* indirect targets. The results showed that 12 *CHD8* direct targets could serve as upstream regulators of 95 of the 841 *CHD8* indirect targets (Fig. 3b), including ASD-risk genes *MEF2* [64] and *ARNT2* [65]. This analysis also revealed that some DEGs could be regulated by 35 DEGs that were, themselves, indirect targets. Together, these 47 upstream regulators formed a complex regulatory network (Fig. 3b), mediating key cellular pathways (e.g., BMP and TGFβ) that eventually may lead to expression changes for thousands of genes when *CHD8* expression is disrupted, an interesting finding to be further investigated.

### *CHD8*-regulated genes are overall longer than non-DEGs

**Fig. 4:**
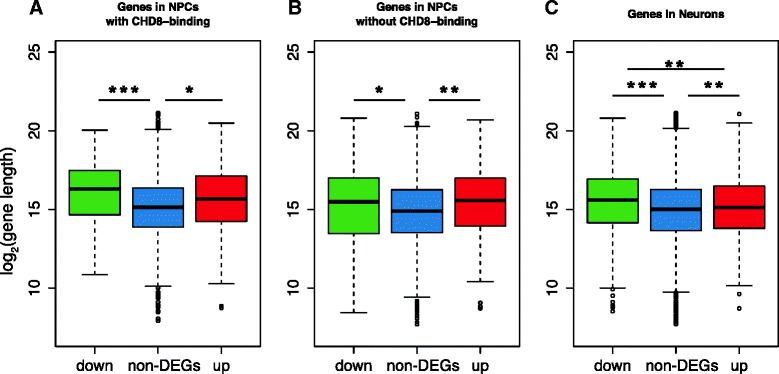
Difference in gene length between DEGs and non-DEGs. Plotted here are length distributions of genes with *CHD8* binding in NPCs (**a**), without *CHD8* binding in NPCs (**b**), and all genes in neurons (**c**). In NPCs, mean of gene lengths is 117 kb for DEG and 76 kb for non-DEG; in neurons, mean of gene lengths is 98 kb for DEGs and 74 kb for non-DEGs, whereas gene lengths were defined as the distances from transcription start site to termination site in Gencode v18. *p < 0.01, **p < 1e-5, ***p < 1e-9, *t* test, two-tailed

We noticed that some extremely long genes were dysregulated in either NPCs or neurons, like *LSAMP* (2187 kb), *PCDH15* (1825 kb), *RBFOX1* (1694 kb), and *NRXN3* (1622 kb). In addition, many high-confidence ASD-candidate genes are exceptionally long [66]. At the genome-wide level, we found that DEGs in both NPCs and neurons were significantly longer than the non-DEGs (Fig. 4). The length difference between DEGs and non-DEGs was not detected in a separate study in which we compared the iPSC-derived neurons from schizophrenia patients with controls (manuscript submitted; data available in the GEO: GSE46562); the mean lengths were 77 kb for DEGs and 73 kb for non-DEGs (*p* value = 0.51, Student’s *t* test). This indicates that our observation is not simply a result of analyzing expression data in the neural induction system we used. This difference was detected for genes with *CHD8*-binding (Fig. 4a) or without *CHD8*-binding (Fig. 4b), suggesting that other transcription regulators may cooperate with *CHD8* to regulate the expression of long genes.

### DEGs are enriched for genes associated with human head size/brain volume

**Fig. 5:**
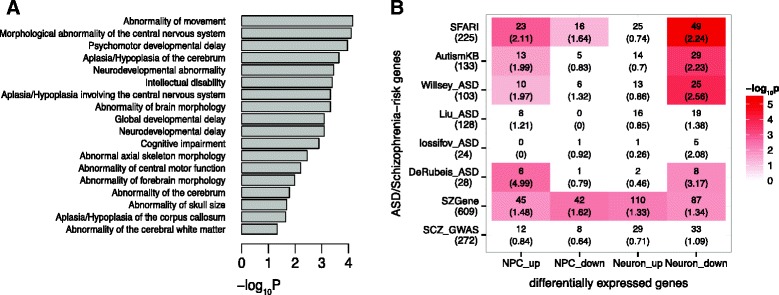
Enrichment of DEGs for ASD/schizophrenia-risk genes and genes associated with specific human phenotypes. **a** Enriched human phenotype from the Toppgene analysis for downregulated genes in neurons. *p* values were corrected by the Bonferroni method. **b** Enrichment of DEGs for ASD- and schizophrenia-risk genes. ASD-risk genes were collected from the SFARI database [54], AutismKB core dataset [55], and four other sets based on whole exome-sequencing and gene-expression network (Willsey_ASD [56], Liu_ASD [57], Iossifov_ASD [11] and DeRubeis_ASD [10]). The number of expressed genes in each set was in left *parentheses*. Schizophrenia-risk genes were obtained from either the SZGene database [58] or a recent GWAS report [59]. The *red color* in each cell corresponds to the −log_10_ (*p* value) for enrichment (Fisher’s exact test, one-tailed), as shown in the *color scale* on the right (shown only if *p* < 0.05). The first and the second number (in *parenthesis*) within each cell are the number of overlapping genes and the odds ratio of overlap, respectively

Macrocephaly is overrepresented in ASD patients and a defined feature of some syndromic forms of ASD, including those carrying loss-of-function *CHD8* mutations [15, 16]. In addition, *chd8* disruption in zebrafish resulted in increased embryonic head size [15, 25]. We thus used Toppgene to predict the potential phenotypes that could result from the dysregulation of *CHD8* targets. For downregulated genes in *CHD8*^*+/−*^ neurons, “abnormality of skull size” was one of the most significant human phenotypes (Fig. 5a), so were neurodevelopmental abnormality, intellectual disability, and abnormality of brain morphology, all consistent with clinical phenotypes seen in patients with *CHD8*-disruptive mutations [15]. For the other gene groups, i.e., up- or downregulated genes in *CHD8*^*+/−*^ NPCs or upregulated genes in *CHD8*^*+/−*^ neurons, either no human phenotypes were significantly enriched, or enriched phenotypes showed no obvious relation to brain or head development (Additional file 6: Table S5). It would be interesting to further study how this difference detected for the DEGs of *CHD8*^*+/−*^ NPCs and neurons may be related to changing *CHD8* roles at different stages of brain development.

Next, we compared the lists of DEGs in *CHD8*^*+/−*^ NPCs and neurons with genes previously associated with brain size or volumes of specific brain regions. Recent GWASs revealed a small number of common variants associated with the volumes of different human brain regions or head sizes [67–69]. Remarkably, of the only 12 genes linked to the statistically significant GWAS variants, 7 were differentially expressed in *CHD8*^*+/−*^ neurons, which was significantly higher than expected (odds ratio (OR) = 4.07, *p* = 0.016, Fisher’s exact test, one-tailed; Table 1). *HMGA2*, a high-confident candidate linked to adult intracranial volume [68] and infant head circumference [69], was a *CHD8* direct target (with *CHD8* binding to its promoter [25, 26]), and it was upregulated in both *CHD8*^*+/−*^ NPCs and neurons. Interestingly, *HMGA2* encodes a chromatin-associated regulator important for stem cell renewal. How *CHD8* and *HMGA2* interact and co-regulate gene-expression and cell cycles warrants further study. Another DEG associated with caudate volume is *FAT3*, which codes for an atypical cadherin previously shown to affect dendritic pruning [70], a known defect in ASD. Interestingly, by analyzing the spatiotemporal transcriptional data across developing brains available in the Brainspan project [71], we found that the expression of *CHD8* was significantly and positively correlated with *FAT3* during brain development (Additional file 2: Figure S2), suggesting that *CHD8* is a positive regulator of *FAT3,* consistent with the downregulation of *FAT3* in *CHD8*^+/−^. *MAPT*, another top candidate associated with infant head circumference [69], was downregulated in *CHD8*^*+/−*^ neuron (nominal *p* value = 0.015).

**Table 1:** Expression changes of genes associated with brain volume or head size from GWAS

Ref.	Trait	SNP	Associated gene	Coordinate	*CHD8*-binding in NPCs	*CHD8* ^+/−^ NPC		*CHD8* ^+/−^ neuron	
						log2(FC)	*q* value	log2(FC)	*q* value
[68]	Hippocampal volume	rs7294919	*TESC*	chr12:117476728-117537284	N	0.42	0.89	*−2.78*	*0.0020*
[68]	Total brain volume	rs10494373	*DDR2*	chr1:162601163-162750237	N	*4.12*	*1.68E-31*	*5.70*	*2.82E-13*
[68]	Intracranial volume	rs10784502	*HMGA2*	chr12:66217911-66360075	Y	*1.00*	*3.57E-06*	*2.97*	*6.67E-08*
[69]	Infant head circumference	rs1042725							
[69]	Infant head circumference	rs7980687	*SBNO1*	chr12:123773656-123849390	Y	−0.15	0.61	*−0.45*	*0.023*
[69]	Infant head circumference	rs11655470	*MAPT*	chr17:43971748-44105700	N	0.61	0.40	−1.82	0.070
			*CRHR1*	chr17:43699267-43913194	N	0.15	0.90	−0.13	0.86
[67]	Intracranial volume	rs17689882	*CRHR1*	chr17:43699267-43913194	N	0.15	0.90	−0.13	0.86
[67]	Putamen volume	rs945270	*KTN1*	chr14:56025790-56168244	Y	−0.22	0.67	−0.09	0.80
[67]	Putamen volume	rs683250	*DLG2*	chr11:83166055-85338966	Y	0.66	0.69	−0.059	0.96
[67]	Caudate volume	rs1318862	*FAT3*	chr11:92085262-92629618	N	*−0.92*	*2.45E-05*	*−1.27*	*4.98E-06*
[67]	Putamen volume	rs6087771	*BCL2L1*	chr20:30252255-30311792	Y	0.10	0.89	*−0.67*	*0.029*
[67]	Hippocampal volume	rs61921502	*MSRB3*	chr12:65672423-65882024	N	0.33	0.79	*2.63*	*8.69E-24*
[67]	Putamen volume	rs62097986	*DCC*	chr18:49866542-51057784	Y	0.72	0.31	−0.18	0.1

Significant fold changes are set in italics

### DEGs are enriched with ASD and schizophrenia-risk genes

Functionally disruptive mutations in *CHD8* have been reported in multiple ASD patients as well as one sporadic schizophrenia patient [72]. Comparing our list of DEGs with ASD-risk gene sets from multiple sources (see Methods), we found that upregulated genes in *CHD8*^*+/−*^ NPCs and downregulated genes in *CHD8*^*+/−*^ neurons were significantly enriched with ASD-risk genes (Fig. 5b, Additional file 7: Table S6). In Table 2, we provided a list of high-confident ASD-risk genes that were dysregulated in *CHD8*^*+/−*^. For the 163 ASD-risk genes that were in our DEG lists, they were significantly enriched for “transmission of nerve impulse,” “synaptic transmission,” and “neuron differentiation,” further supporting the importance of *CHD8* in regulating synaptic functions. In a comparison of our DEGs with the genes associated with schizophrenia, we found that our DEGs were enriched with schizophrenia-related genes from the SCZgene database [58] but not enriched in the high-confident gene list from a recent schizophrenia GWAS [59] (Fig. 5b, Additional file 7: Table S6).

**Table 2:** Selected differentially expressed genes associated with ASD risk

Gene	Coordinate	ASD score			*CHD8*-binding in NPCs	*CHD8* ^+/−^ NPC		*CHD8* ^+/−^ neuron	
		SFARI	AutismKB	Willsey		log2(FC)	*q* value	log2(FC)	*q* value
*ANK2*	chr4:113739265-114304896	High confidence	9	hcASD	N	*−1.87*	*1.36E-45*	*−1.96*	*6.32E-08*
*SETD5*	chr3:9439299-9520924	High confidence	10		Y	0.25	0.67	*0.87*	*0.00053*
*SUV420H1*	chr11:67922330-67981295	High confidence	16	hcASD	Y	−0.084	0.89	*−0.86*	*0.0020*
*SCN2A*	chr2:166095912-166248818	High confidence	20	hcASD	N	−0.64	0.86	*−3.31*	*5.67E-05*
*DEAF1*	chr11:644233-706715	Strong candidate	2		N	−0.16	0.79	*−0.68*	*0.0010*
*MYT1L*	chr2:1792885-2335032	Strong candidate	2		N	0.67	0.72	*−1.76*	*0.0077*
*BCL11A*	chr2:60678302-60780702	Strong candidate	9		N	*1.91*	*0.029*	0.25	0.61
*CNTN4*	chr3:2140497-3099645	Strong candidate	9		N	6.02	0.53	*5.15*	*1.60E-14*
*CACNA2D3*	chr3:54156574-55108584	Strong candidate	10	pASD	Y	*2.64*	*0.0036*	*−2.95*	*0.00076*
*CACNA1H*	chr16:1203241-1271771	Strong candidate	10		Y	*0.75*	*0.045*	−0.19	0.58
*NRXN1*	chr2:50145643-51259674	Strong candidate	28	pASD	Y	−0.81	0.77	*−1.71*	*2.13E-05*
*MET*	chr7:116312444-116438440	Strong candidate	33		N	0.69	0.82	*−3.56*	*3.45E-05*
*GABRB3*	chr15:26788693-27184686	Strong candidate	34		N	−0.43	0.66	*−1.70*	*0.00030*
*RELN*	chr7:103112231-103629963	Strong candidate	43	pASD	N	1.82	0.16	*−2.92*	*1.46E-06*

Significant fold changes are set in italics

### *CHD8*-regulated genes significantly overlap with the targets of other autism-risk genes

Since ASD is caused by mutations in a diverse array of genes involved in different cellular functions, we next set out to address whether the dysregulated targets by different ASD-risk genes indeed converge on molecular and cellular pathways. We thus searched for published expression data reporting downstream targets of ASD genes, especially transcriptional regulators. Recent studies reported that in human progenitor cells, reduced expression of transcription factor 4 (*TCF4*) and histone-lysine N-methyltransferase 1 (*EHMT1*) converged at several levels with respect to their downstream targets [51]. Also, the downstream targets of methyl-CpG-binding domain 5 (*MBD5*) and special AT-rich binding protein (*SATB2*) were converged in neural stem cells [52]. We therefore decided to include these genes to our analysis because their association with ASD has been described previously [8, 73, 74], and their regulatory targets were identified in NPCs using an experimental scheme similar to ours, comparing expression changes by RNA-seq after reducing expression of ASD-candidate genes that function as transcription regulators.

**Fig. 6:**
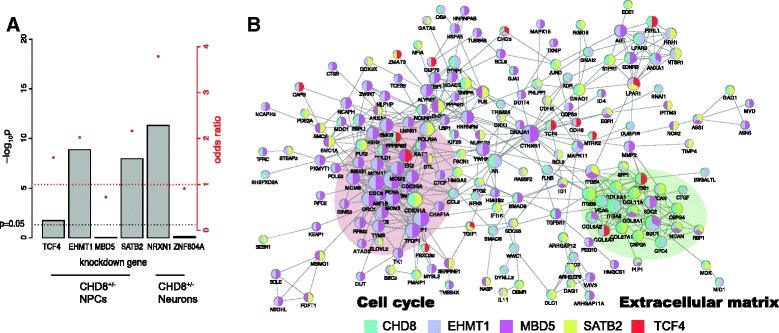
Overlap between DEGs from *CHD8* ^*+/−*^ and previous knockdown studies of other ASD-risk genes. **a** Enrichment test for the overlaps between DEGs from *CHD8* ^*+/−*^ NPCs and knockdowns of *TCF4*, *EHMT1*, *MBD5*, or *SATB2*, and between DEGs from *CHD8* ^*+/−*^ neurons and knockdowns of *ZNF804A* or *NRXN1. Bars* represent *p* value (Fisher’s exact test, one-tailed), *red dots* represent odds ratio of overlap. **b** Network analysis of genes that were differentially expressed in NPCs in either *CHD8* ^*+/−*^ or knockdown of at least two of the five genes (*CHD8*, *TCF4*, *EHMT1*, *MBD5*, and *SATB2*). The network was generated by STRING, with *nodes* representing genes and *edges* representing interactions. *Size* of the *nodes* is proportional to their connectivity. *Colors* of the *nodes* label the sources of DEGs. Two natural clusters are demarcated with * ellipses*. Disconnected genes were not shown

First, we compared the DEGs from these studies with ours. Note that in *CHD8*^*+/−*^ NPCs, *TCF4* was upregulated (3.4-fold increase, *q* value = 1.58e-19), but no expression changes were observed for the other three. We found that our list of DEGs in NPCs showed significant overlap with the DEGs found in the *TCF4*, *EHMT1*, and *MBD5* knockdown studies (Fig. 6a, Additional file 8: Table S7). To search for functional commonality, we analyzed the 439 genes that were affected by at least two of the five ASD genes. Again, we used STRING to define function interactions among these 439 genes. The results indicated that the common genes were mainly distributed in two highly interconnected clusters (Fig. 6b). One was significantly enriched for genes involved in forming extracellular matrix (*p* = 5.83e-12, Additional file 8: Table S7), including multiple collagen genes. The other was highly enriched with cell cycle-related genes (*p* = 6.78e-9, Additional file 8: Table S7), though the enrichment was mainly derived from the common genes between *EHMT1* and *MBD5* knockdown. Genes critical for neurogenesis, like *PLP1* and *GFAP*, were also significantly enriched (*p* = 1.14e-8, Additional file 8: Table S7), but they showed sparse connection, perhaps because of incomplete information in the interaction database.

Next, we carried out the same analysis for DEGs in neurons, including data from our current study and a previous study in which neurexin 1 (*NRXN1*) expression was reduced [75]. *NXRN1* was downregulated in *CHD8* knockout neurons (3.2-fold reduction, *q* value = 2.84e-4). Again, we found a significant overlap of the DEGs from *CHD8* knockout neurons with those in *NRXN1* knockdown neurons (Fig. 6a). The overlapping genes were, again, enriched for “extracellular matrix.”

The transcriptional targets of zinc finger protein 804A (*ZNF804A*), a top schizophrenia candidate, were recently reported by our group from RNA-seq analysis in which the gene was knocked down in neurons derived from iPSCs [49]. While the overlap of DEGs from differentiating neurons with *CHD8* knockout and *ZNF804A* knockdown was not significant (Fig. 6a), the overlap of DEGs from *ZNF804A* knockdown and *NRXN1* knockdown was highly significant (OR = 2.63, *p* = 0.023, Fisher’s exact test, one-tailed). These results suggest that both common and unique transcription targets exist for neuropsychiatric disorder candidate genes that code for gene-expression regulators. It should also be noted that previous studies have also reported a convergence of genes affected by *SATB2* knockdown and *MBD5* knockdown [52].

In summary, our comparison of genes that show expression changes upon knockout or knockdown of key genes implicated in major neuropsychiatric disorders found that many downstream target genes were commonly affected by several transcription factors and epigenetic modifiers, and the common genes were often enriched for those that code for “cell cycle,” “extracellular matrix,” and “cell proliferation.”

### Comparison with previous results from *CHD8* knockdown studies

We compared our lists of DEGs with other four lists of DEGs that were obtained by knocking down *CHD8* expression in neural cells [25–27]. Except for the two lists from two independent shRNA knockdowns in the same study, DEG lists from different analyses showed modest overlap; at most, 30–40 % of DEGs in one list were detected in other studies (Additional file 2: Figure S3).

**Fig. 7:**
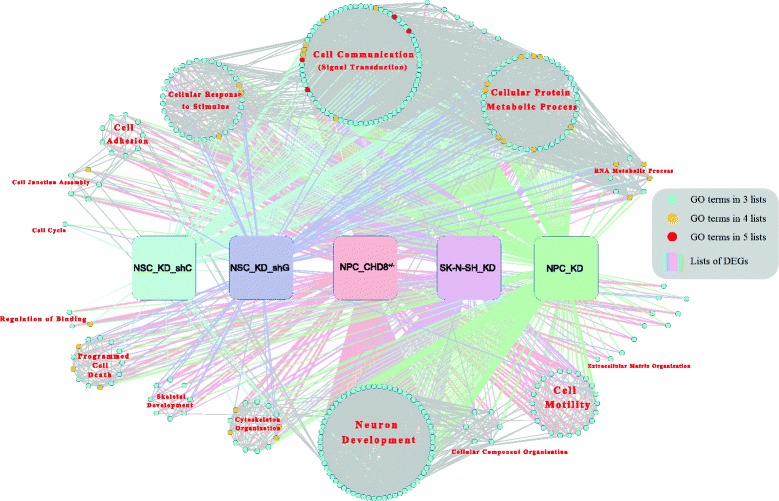
Enriched GO terms shared among five lists of DEGs from four studies. DEGs were from *CHD8* ^+/−^ NPC (current data), NPC knockdown (NPC_KD) [25], NSC knockdown by two independent shRNAs (NSC_KD_shC, NSC_KD_shG) [26], or SK-N-SH knockdown (SK-N-SH_KD) [27]. *Nodes* represent common GO terms (names not displayed) and *grey edges* between them depict the degrees of gene overlap between terms. GO Terms enriched in 3, 4, and 5 DEG lists are colored *blue*, *orange*, and *red*, respectively. Individual GO terms with a similar gene composition were clustered into functional groups by ClueGO and drawn together as *circles*, with the functions labeled in *red font. Square boxes* represent each of the five DEG lists and *colored edges* connect enriched GO terms in individual studies. This visualization was created using ClueGO plugin in the Cytoscape

Whereas the overlap from different studies was modest, we considered the possibility that the DEGs from different studies might converge on common functional pathways. We thus performed GO enrichment analysis for each of the five lists of DEGs and then focused on the 327 GO terms that were enriched in at least three DEG lists (Additional file 9: Table S8). Among them, only four terms were present in all five DEG sets (referred to as “5 L” terms) and 31 terms (“4 L” terms) were shared by four studies. When the functions of these 327 GO terms were grouped by the software GlueGO [61], however, 13 major functional clusters emerged (Fig. 7). The largest cluster, harboring 72 individual GO terms, was involved in cell communication, to which all of the 5 L terms belonged. This suggests that one common effect of reduced *CHD8* expression across all studies is cell communication and signal transduction. Some of the DEGs from our *CHD8*^*+/−*^ analysis are regulators of major signaling pathways, for example, *NFATC1* and *BMP2/4*, and *WNT7A* (Fig. 3b). This finding was further supported by the observation that 62 % of the 4 L terms (*n* = 22) were located within similar function clusters (indicated by high density of grey lines between them), such as cellular protein metabolic process, RNA metabolic process, and cellular response to stimulus. The second largest cluster was “neuron development,” including 66 specific GO terms related to neuron differentiation, axon guidance, and synapse organization, etc. This indicates that *CHD8* disruption has a profound effect on multiple aspects of neurodevelopment. Note that the two DEG lists from shRNA knockdown in the study by Cotney et al. were not enriched for this large functional cluster. However, cell cycle pathways were particularly enriched in those two lists, suggesting that the samples in that study might be in a more proliferative and less “neural-like” state. This integrated analysis also identified a number of GO terms clustered into programmed cell death and cytoskeleton organization, consistent with the previously reported interactions between *CHD8* and p53-mediated apoptosis [23] and the Wnt-β-catenin signaling pathway [19]. In addition to well-known functional clusters involving *CHD8,* including cell adhesion and extracellular matrix organization, our bioinformatics analysis suggests that cell migration and skeletal development could also be a common theme affected by reduced *CHD8* expression.

**Fig. 8:**
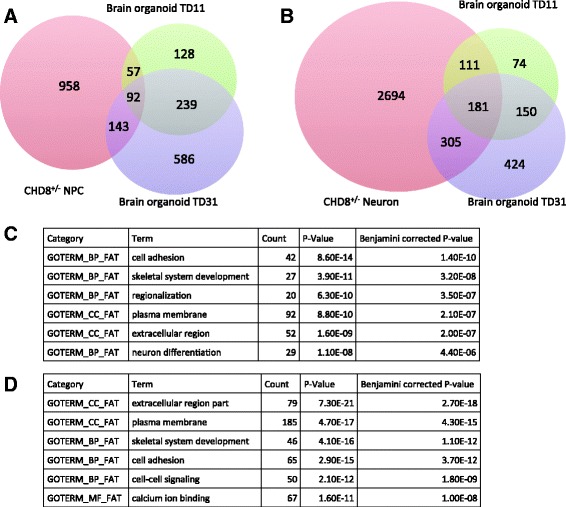
Comparison of DEGs from *CHD8* ^*+/−*^ and DEGs from brain ASD organoids [76]. Venn diagrams of DEGs from *CHD8* ^*+/−*^ NPCs (**a**) or neurons (**b**) and DEGs from brain organoids. The *number* in each section represents genes that are also expressed in our samples. Top-enriched GO terms for overlapped genes between DEGs from *CHD8* ^*+/−*^ NPCs (**c**) or neurons (**d**) and DEGs from brain organoids referred to as TD11 and TD31

Finally, we compared the five lists of DEGs to the results in a recent analysis of telencephalic organoids generated from idiopathic autism patient-specific iPSCs [76]. Of the differentially expressed genes in two developmental stages (TD11 and TD31) of the organoids between ASD and control samples [76], 516 and 1060 were expressed in our samples. When the DEGs in *CHD8*^*+/−*^ or *CHD8* knockdown studies were compared to the organoid DEGs, our *CHD8*^*+/−*^ showed the largest overlap with the organoid data (Fig. 8, Additional file 2: Figure S3). GO analysis indicated that the dysregulated genes common in *CHD8*^*+/−*^ samples and ASD organoids were significantly enriched for genes involved in skeleton system development, cell adhesion, and neuron differentiation (Fig. 8), indicating that these could be functional pathways commonly disrupted in ASD. Interestingly, the brain volume-associated gene *FAT3* was upregulated in TD31, further suggesting the potential importance of *FAT3* in ASD.

## Discussion

To better understand the effect of genetic disruption of *CHD8* in ASD subjects, we have applied CRISPR/Cas9 technology to knockout one copy of *CHD8* in a control iPSC line and studied its effect on gene expression during early neurodevelopment. In comparison to previous studies [25–27] that used a gene knockdown approach to reduce *CHD8* expression, our approach generated heterozygous disruptions that better mimic the germline mutations in ASD patients and allows for the study of long-term effects of *CHD8* disruption in neurogenesis in vitro. In addition, creating *CHD8*^*+/−*^ iPSCs provides a truly renewable resource for investigators, as opposed to NPCs, which have a finite replicative capacity. Furthermore, iPSCs can differentiate into any cell type, including cerebral organoids [77]; NPCs are restricted in their differentiation potential.

Perhaps one of the most important findings emerging from our transcriptomic analysis is that several genes known to be related to head size or brain volume, either from the analysis of human phenotype ontology [46] or identified through GWAS [67–69], displayed significant changes in their expression in *CHD8*^+/−^ neurons. Studies examining *CHD8* function in zebrafish during embryonic development revealed that the macrocephaly phenotype observed in ASD probands with *CHD8* mutations is likely caused by disturbed neuronal proliferation at early developmental stages [15, 25]. By uncovering genes like *HMGA2* and *FAT3*, which have been associated with head size, our finding thus provides new molecular insights that may eventually link *CHD8* mutation to abnormal neuronal proliferation and macrocephaly. This association between macrocephaly and ASD is not the first to be reported in genetically defined subgroups. Mutations in *PTEN* are also associated with severe macrocephaly and ASD [78]. Although *PTEN* itself was not differentially expressed in our *CHD8*^*+/−*^ samples, *FOXO1* and *FOXO3*, two critical transcription factors in PTEN signaling, were. Interestingly, IPA analysis of the differentially expressed genes reported an enrichment of genes in PTEN signaling, suggesting that there may be a link between the PTEN and *CHD8* pathways, and the molecular link underlies the observed macrocephaly and ASD. In this regard, we should mention that *PTEN* was differentially expressed upon *CHD8* knockdown in NPCs in a previous study [25]. We should also point out there were additional genes in our DEG lists that have been previously suggested to be associated with hippocampal volumes, such as *BDNF*, *DISC1*, and *NRG1.*

Dysregulated genes in *CHD8*^*+/−*^ cells exhibited significant overlap with previously defined ASD-risk genes, including some high confident candidates like *ANK2*, *SCN2A*, and *SUV420H1* [54] that also showed significant differential expression (Table 2). We further demonstrated, interestingly, that *CHD8*-regulated genes significantly overlapped with the downstream targets of other ASD-risk genes like *TCF4* and *NRXN1*, providing transcriptomic evidence that ASD-risk genes have overlapping function and converge on downstream regulatory pathways. This is extremely important in a genetically heterogeneous condition, such as ASD, in which any individual candidate gene carrying deleterious mutation may only contribute to 1~2 % ASD cases [79]. *TCF4* is associated with Pitt-Hopkins syndrome (MIM: 610954), which is defined by severe psychomotor delay, epilepsy, daily bouts of diurnal hyperventilation, mild postnatal growth retardation, postnatal microcephaly, and distinctive facial features [80]. It is also a top schizophrenia candidate gene. In NPCs and NSCs, *CHD8* binds to the *TCF4* gene body in a region that is also enriched with H3K27ac [25, 26], a histone modification associated with active enhancers. *TCF4* is significantly upregulated in both *CHD8*^+/−^ NPCs and neurons. Moreover, TCF4-regulated genes significantly overlapped with *CHD8*-regulated genes. Taken together, these results suggest a direct connection between *TCF4* and *CHD8* regulatory networks. The common genes in the two networks, especially those regulated oppositely by *CHD8* and *TCF4*, such as *HMGA2*, which was upregulated in *CHD8*^*+/−*^ (Table 1) and downregulated in *TCF4* knockdown [81], are strong candidates for regulating the development of brain size.

It was intriguing to find that DEG lists from different *CHD8*^*+/−*^ or knockdown studies had only limited overlaps. A recent study of knockout vs. knockdown in zebrafish *egfl7* proposed that compensatory networks would be activated to buffer against deleterious mutations from knockout, which was absent in knockdown [82], providing a potential explanation for the lack of good overlap among *CHD8* KO and KD findings. However, we found that at the function and pathway levels, genes involved in similar functions were affected by reduced *CHD8* expression in different contexts. It is conceivable that a limited number of upstream regulators are directly regulated by *CHD8*, and the subsequent response of the downstream targets is mostly dependent on genetic background, cell culture conditions, and other experimental factors. In this regard, we should mention that five (*GDPD4*, *VPS13B*, *KMT2C*, *SETBP1*, and *CLTCL1*) of the ~1000 ASD-risk genes were predicted to be functionally disrupted by premature stop or frameshift variants located to the coding exons of the subject used to prepare our WT iPSC line (Additional file 10: Table S9 and S10). However, none of these five genes exhibited differential expression in the WT neurons when compared to samples from other control iPSC lines derived from six unrelated subjects (data not shown). As neuronal differentiation is a complex process, affected by both environmental cues and intrinsic cellular signaling, our analysis suggests that it is important to study the effects of the same genes under different experimental conditions. Nevertheless, comparison of our results with the transcriptomic data in ASD-derived organoids indicates that reduction of *CHD8* expression in our *CHD8*^*+/−*^ samples is probably more consistent with the gene regulation in ASD-developing brains, although it should be noted that the ASD-derived organoids were from patients with unknown genetic mutations.

As most of the functionally disruptive mutations uncovered in the *CHD8*-coding regions in the ASD probands introduce premature stop or frameshift mutation [15, 16], we used CRISPR/Cas9 technology to make small deletions in the first coding exon of *CHD8* in this study. While the 2- or 10-bp deletion is predicted to cause frameshift, and no functional protein is expected from the mutants, RNA transcripts from the knockout *CHD8* copy were observed in our RNA-seq data, indicating nonsense mediated mRNA decay is incomplete if it occurs to the mutated *CHD8* transcripts (Fig. 1). *CHD8* encodes a multi-domain protein, and deleterious mutations in *CHD8* have been found in almost every important functional domain [15, 16]. While our data indicate that *CHD8* regulates multiple pathways related to neural development, the different mutations in ASD individuals may impair distinct and specific aspects of *CHD8* functions. In the future, it will be valuable to carry out gene-expression profiling using ASD-specific iPSCs to see how our current findings can be recapitulated in additional iPSC-derived NPCs and neurons. As genetic background likely plays an important role in modulating *CHD8* function during brain development, it is important to both perform our current knockout analysis in additional control iPSC lines and to derive patient-specific lines from multiple ASD individuals with *CHD8* mutations. In addition, it will be extremely valuable to apply CRISPR technology to correct the *CHD8* mutations and perform transcriptomic analysis and other molecular assays once the patient-specific lines are established.

## Conclusions

*CHD8* regulates multiple genes involved in cell communication, extracellular matrix and neurogenesis that are critical for brain development. In addition, *CHD8* hemizygosity causes expression changes in several genes that are associated with brain volume or head size, suggesting a molecular link between *CHD8* mutation and macrocephaly. By cross-analysis of several studies in which the expression of several ASD-candidate genes was reduced, we provide evidence that the transcription targets of ASD genes converge on a set of genes and pathways.

## Additional files

Additional file 1:
**Supplemental methods.** (PDF 101 kb) Additional file 2:
**Figure S1–S3 and Table S1.** (PDF 367 kb) Additional file 3:
**Table S2.** Full list of gene-expression values, fold changes, and statistics. (XLSX 562 kb) Additional file 4:
**Table S3.** GO analysis (DAVID) of DEGs in NPCs and neurons (only terms with Benjamini corrected *p* < 0.05 were included). (XLSX 155 kb) Additional file 5:
**Table S4.** Pathway analysis (IPA) of DEGs in NPCs and neurons (only pathways with Benjamini corrected *p* < 0.05 were included). (XLSX 177 kb) Additional file 6:
**Table S5.** Human phenotype analysis (Toppgene) of DEGs. (XLSX 153 KB) Additional file 7:
**Table S6.** DEGs associated with ASD or schizophrenia. (XLSX 49 kb) Additional file 8:
**Table S7.** DEGs from *CHD8* ^*+/−*^ or knockdown of *TCF4*, *EHMT1*, *MBD5*, *SATB2*, *NRXN1*, or *ZNF804A* expression (“1” represents differential expression, “0” represents no change) and GO analysis from the STRING (only term with FDR < 0.05 were included). (XLSX 292 kb) Additional file 9:
**Table S8.** GO analysis (ClueGO) of DEGs in each of five DEG lists from *CHD8* ^*+/−*^ or knockdown analysis. (XLSX 670 kb) Additional file 10:
**Table S9–S10.** Numbers and putative functionally disrupted coding variants in the WT iPSC line. (PDF 34 kb)

## Abbreviations

*CHD8*: Chromodomain helicase DNA binding protein 8
CNV: Copy number variation
DEG: Differentially expressed gene
FPKM: Fragments per kilobase of exon per million fragments mapped
GO: Gene ontology
GWAS: Genome-wide association study
IPA: Ingenuity pathway analysis
iPSC: Induced pluripotent stem cell
KD: Knockdown
KO: Knockout
NPC: Neural progenitor cell
NSC: Neural stem cell
OR: Odds ratio
sgRNA: Single guide RNA
WT: Wild-type
