# Supplementary material for: CRISPR/Cas9-mediated heterozygous knockout of the autism gene CHD8 and characterization of its transcriptional networks in neurodevelopment
Source: Mol Autism. 2015 Oct 19;6:55. doi: 10.1186/s13229-015-0048-6 (PMC4612430; doi:10.1186/s13229-015-0048-6)
Supplement: Additional file 1: — Supplemental methods. (PDF 101 kb) [file 13229_2015_48_MOESM1_ESM.pdf]

Supplemental methods

Subjects used for iPSC development. The *CHD8*$^{+/-}$ lines were generated in an iPSC line from a control subject who was recruited at the Albert Einstein College of Medicine as part of a larger study to generate iPSCs from control subjects and patients with psychosis who have a 3Mb deletion on 22q11.2. The subject signed a written consent approved by the Albert Einstein College of Medicine IRB (Institutional Review Board). Fibroblasts were obtained from a punch skin biopsy performed by a board-certified dermatologist.

Harvesting Fibroblasts. Skin biopsy samples were transferred to a small Petri dish containing 2-3 ml Skin Fibroblast Media (SFM) consisting of RPMI 1640, 10% FBS, 1% pen/strep, 10ng/ml FGF2. The sample was incubated at room temperature for 15 minutes. Medium was carefully aspirated and replaced with 1-2 ml of collagenase type II solution (3mg/ml collagenase II dissolved in DMEM High Glucose [Worthington Biochemical Corp. Lakewood, NJ: GIBCO/Invitrogen, Carlsbad, CA]). The tissue was chopped into small pieces using 2 sterile scalpels, after which they were allowed to incubate at 37°C for 1-2 hours depending on size. The sample was then collected in a 15ml falcon tube and washed with SFM (serum free medium). Tissue was collected by centrifugation at 100G for 4 minutes. The pelleted sample was then suspended in SFM and plated in a T12.5 ml flask at 37°C in 5% CO_2 for 3 days without changing medium or manipulation, to allow fibroblasts to adhere. Then cells were subsequently fed every 2 days with RPMI 1640 containing 10% FBS until a confluent culture was obtained (~3 weeks).

Establishing Human iPSCs. iPSC reprogramming was carried out by nucleofection [1]. One vial of cells was thawed out and placed in a T75 flask in DMEM/F12 supplemented with 10% FBS and fed every 2 days. Cells were grown to ~50% confluence (~4-5 days), after which they were trypsinized and subjected to nucleofection (~6 x10^5 cells). Reprogramming was carried out using an Amaxa 4D-Nucleofector (P2 Primary Cell Kit from Lonza cat# V4XP-2012, Program FF-135) with non-integrating plasmids containing OCT4, SOX2, KLF4, L-MYC, LIN28, and a p53 shRNA vector (Addgene Cat. # 27077, 27078, 27080), according to Okita et al., with some modifications [1-3]. iPSCs were maintained on Matrigel plates in mTeSR1 medium (Stem Cell Technologies) with daily feeding in 37°C/5% CO_2/85% humidity.

Germ line markers, establishing pluripotency by *in vitro* differentiation and karyotype. Pluripotency for all iPSC lines was confirmed by immunocytochemistry using antibodies (Ab) against Tra-1-60, Tra-1-81, SSEA3 and SSEA4, which are expressed in pluripotent stem cells. In addition, the capacity to differentiate into all 3 germ layers was established by *in vitro* assays, as previously described [3, 4]. The markers desmin (mesoderm), α-fetoprotein (endoderm), and βIII-tubulin (ectoderm) were used. A list of the Ab used in the study is shown below. Karyotyping was carried out by Cell Line Genetics (Madison WI). All lines had normal karyotypes.

CRISPR-cas9 knockout. Human iPSCs were cultured and fed daily in mTeSR1 (Stem Cell technologies) on Matrigel (BD) coated plates at 37°C/5% CO_2/85% in a humidified

incubator. Cells were maintained in log phase growth and differentiated cells were manually removed before starting the experiment. iPSCs were exposed to 10uM ROCK Inhibitor for ~4 hours to ensure cell survival during nucleofection. After 4 hours, growth medium was aspirated and the cells were rinsed with DMEM/F12. iPSCs were dissociated into single cells via accutase and harvested. Nucleofection was performed using the Amaxa-4D Nucleofector Basic Protocol for Human Stem Cells (Lonza) according to the manufacturer's instructions. Briefly, $8x10^5$ cells and 5ug of plasmid were nucleofected using the P3 Primary Cell 4D-Nucleofector X Kit L with program CA-137. Cells were resuspended in mTeSR1 + 10uM ROCK Inhibitor and placed in one well of a 6-well Matrigel-coated plate. The following day, cells were fed with fresh mTeSR1, and were subsequently fed with fresh medium every day. On days 4-14, cells were exposed to 0.5ug/ml puromycin for 6 hours. Puromycin-resistant colonies were were picked and expanded in mTeSR1 without further puromycin treatment.

Neuronal differentiation. iPSCs were maintained in mTeSR1 medium (Stem Cell Technologies) for approximately 5-6 days. Colonies were checked for spontaneous differentiation under a dissecting microscope; clusters of differentiated cells were removed manually. The medium was then changed to N2 (DMEM/F12, 1X N2; Invitrogen). After 24 hours, medium was changed to N2 plus 1μM Dorsomorphin (CALBIOCHEM). The following day, embryoid bodies (EBs) were created. Briefly, iPSCs were checked again for spontaneous differentiation and fresh N2 medium plus 1μM Dorsomorphin was added. Colonies were cut with a 5ml glass serological pipet using wide strokes to generate large fragments. A cell scraper was then used to detach remaining cells. EBs were aliquoted to a 6-well, ultra-low attachment plate (Corning). Two days later, EBs were collected in a 15ml tube and allowed to settle by gravity for 5 minutes. Supernatant was removed and fresh N2 media plus 1μM Dorsomorphin was added. EBs were aliquoted to a new ultra-low attachment plate. From this point, EBs were fed every other day for 6 days, after which neuronal differentiation was induced. Neural progenitor cells (NPCs) were generated from neural rosettes as previously described by Marchetto et al. with slight modifications [2, 3, 5]Briefly, EBs were collected in a 15ml tube and allowed to settle by gravity for 5 minutes. Supernatant was removed and EBs were gently resuspended in NBF medium (DMEM/F12, 0.5X N2, 0.5X B27, 1% p/s) plus fresh 20ng/ml FGF2 (R&D Systems). EBs were gently aliquoted to a matrigel (BD Biosciences) plate using a 10 ml pipet. Two days later, plates were checked for rosette formation and fed with NBF medium plus fresh FGF2. Rosettes were fed every other day x 2, then carefully excised with a 26g needle and pooled in a 1.5ml tube. Accutase (ICT) was added to the rosettes for 3 minutes at 37^oC. After incubation, rosettes were broken up with a 1ml pipet tip, centrifuged for 2 minutes at 100G, and washed once with 1X PBS (Invitrogen). The pellet was resuspended as single cells in NBF media with fresh FGF2 (20ng/ml) and aliquoted onto Poly-L-Ornithine (Sigma)/Laminin (Roche) plates. NPCs were fed every other day. Once NPCs reached ~50% confluence, neural differentiation was initiated by withdrawing FGF2 and adding NBF media supplemented with fresh growth factors as follows: WNT3A (100ng/ml) (R&D Systems), BDNF (10ng/ml), GDNF (10ng/ml), IGF-1 (10ng/ml) (PeproTech), and cAMP 1μM (Sigma). Cells were fed every other day. The protocol produces a heterogeneous mix of glutamatergic neurons and GABAergic neurons (~50-50 mix).

Characterizing *CHD8*$^{+/-}$ lines

TA" cloning was used to identify the knockout alleles. A 479 bp PCR amplicon flanking the CRSPR/cas9 targeted sites was generated using the primers 5'-CTGTAAGACAGGTTGGGCTG-3' and 5'-CTTGTTTCTTGCCTCTATACTTGA-3'. The PCR product was purified and ligated into pCR™2.1 using a TA Cloning Kit developed by Life Technologies following the manufacturer's protocol. Recombinant plasmids were introduced into competent E. coli and selected in ampicillin. Plasmid DNA was extracted and sequenced across the insert by using one of the PCR primers. Western blotting was used to confirm that the KO lines expressed lower levels of CHD8 protein

Reverse transcribed PCR (RT-PCR) and quantitative real-time PCR (qPCR)

Total RNA was extracted using a miRNeasy Kit according to the manufacturer's instructions (Qiagen). An additional treatment with DNase1 (Qiagen, Valencia, CA) was included to remove genomic DNA. Reverse transcribed PCR (RT-PCR) was performed using a OneStep RT-PCR Kit (Qiagen, Valencia, CA) according to the manufacturer's instructions. The cDNA was used as a template for quantitative PCR (qPCR), which was carried out using the ABI 7900HT Real-Time PCR System (Applied Biosystems, Foster City, CA). Each reaction consisted of cDNA, primers, and SYBR Green PCR Master Mix (Applied Biosystems, Foster City, CA) in an 8 µl volume. Melting curve analysis of target sequences showed that all primers used in this study generated amplicons that had a single peak, without primer-dimer artifacts. Primer concentrations were optimized prior to use in qPCR experiments. Relative changes in gene expression were calculated using the $2^{-\Delta\Delta Ct}$ method with β2-microglobulin (β2M) as a reference gene. Each qPCR was carried out in triplicate, with each triplicate data point repeated 3 times. For the triplicates, only samples that differed by <0.3 Ct values were used in the final calculations. Less than ~5% of samples fell out of this range. In addition, standard curves were generated for each gene using a 50-fold dilution range. qPCR experiments were only used in the final analysis if the slope of the Ct vs input curve was at least -3.0 and the correlation coefficient for triplicate sames was >0.98. Relative changes in gene expression were calculated using the $2^{-\Delta\Delta Ct}$ method with β2-microglobulin (β2M) as a reference gene. Significant differences in gene expression were assessed using a two-tailed student's T-test.

PCR primers used in this study

Gene	Forward	Reverse
**Characterizing iPSCs**		
OCT4 plasmid	CATTCAAACTGAGGTAAGGG	TAGCGTAAAAGGAGCAACATAG
KLF4 plasmid	CCACCTCGCCTTACACATGAAGA	GCGTAAAAGGAGCAACATAG
SOX2 plasmid	TTCACATGTCCCAGCACTACCAGA	TTGTTTGACAGGAGCGACGAT
L-MYC plasmid	GGCTGAGAAGAGGATGGCTAC	TTTGTTTGACAGGAGCGACGAT
LIN28 plasmid	AGCCATATGGTAGCCTCATGTCCGC	TAGCGTAAAAGGAGCAACATAG
β2M	GCTCGCGCTACTCTCTCTTT	CAATGTCGGATGGATGAAAC
**qPCR**		
SMARCA2	GGCTTCTTTTGTCACCCTGA	CACCAACACCACATTCTTCG

HMGA2	GGTGCCACCCACTACTCTGT	TGAGATTGAAAGTGCCTTGG
TESC	TTGAGACCAAGATGCACGTC	TTGCAAAGTGCAGTTTCTCC
DDR2	CGACGAGTGATGCTGTCAGT	TGTCCAGATGGAGTGGCATA
WNT7A	CCCACCTTCCTGAAGATCAA	GTCCTCCTCGCAGTAGTTGG
TCF4	CTGAAAGCTGCTCGCTAGGT	AAAGGCCACATTCCCTTTTT
TGFB3	GGATCACCACAACCCTCATC	CCGGAAGCAGTAATTGGTGT

Antibodies used in this study

Antibody	Company	Catalog #
Anti-human Tra 1-60	eBioscience	12-8863-80
Anti-human Tra 1-81	eBioscience	12-8883-80
AF488 Anti-mouse/human SSEA-3	eBioscience	53-8833-71
AF488 Mouse anti SSEA-4	BD Pharmingen	560308
Anti-Tubulin, beta III isoform	Millipore	MAB1637
Desmin Ab-1	ThermoScientific	MS-376-S
Anti-human/mouseα-Fetoprotein	R & D	MAB1368
PSD95 (mouse)	UC Davis/NIH NeuroMab Facility	75-028
Synaptophysin(rabbit)	Abcam	ab8049
Anti-GAD65/67	Sigma	G5163
Ms anti- Vglut2	Millipore	MAB5504
Rabbit neuronal class III β-tubulin	Fisher	NC9168644
Tbr1	Abcam	Ab31940
Sheep anti-Tyrosine Hydroxylase	Pel-Freez	P60101

References

1. Okita K, Matsumura Y, Sato Y, Okada A, Morizane A, Okamoto S, Hong H, Nakagawa M, Tanabe K, Tezuka K, Shibata T, Kunisada T, Takahashi M, Takahashi J, Saji H, Yamanaka S: A more efficient method to generate integration-free human iPS cells. Nat Methods 2011; 8: 409-12

2. Chen J, Lin M, Hrabovsky A, Pedrosa E, Dean J, Jain S, Zheng D, Lachman HM: ZNF804A Transcriptional Networks in Differentiating Neurons Derived from Induced Pluripotent Stem Cells of Human Origin. PLoS One 2015; 10: e0124597

3. Pedrosa E, Sandler V, Shah A, Carroll R, Chang C, Rockowitz S, Guo X, Zheng D, Lachman HM: Development of Patient-Specific Neurons in Schizophrenia Using Induced Pluripotent Stem Cells. J Neurogenet 2011

4. Chen J, Lin M, Foxe JJ, Pedrosa E, Hrabovsky A, Carroll R, Zheng D, Lachman HM: Transcriptome Comparison of Human Neurons Generated Using Induced Pluripotent Stem Cells Derived from Dental Pulp and Skin Fibroblasts. PLoS One 2013; 8: e75682

5. Marchetto MC, Carromeu C, Acab A, Yu D, Yeo GW, Mu Y, Chen G, Gage FH, Muotri AR: A model for neural development and treatment of rett syndrome using human induced pluripotent stem cells. Cell 2010; 143: 527-39
