# Supplementary material for: CRISPR/Cas9-mediated heterozygous knockout of the autism gene CHD8 and characterization of its transcriptional networks in neurodevelopment
Source: Mol Autism. 2015 Oct 19;6:55. doi: 10.1186/s13229-015-0048-6 (PMC4612430; doi:10.1186/s13229-015-0048-6)
Supplement: Additional file 2: — Figure S1–S3 and Table S1. (PDF 367 kb) [file 13229_2015_48_MOESM2_ESM.pdf]

Figure S1: GO term enrichment analysis (DAVID) for genes that were differentially expressed genes between NPCs and neurons in WT but not in *CHD8*$^{+/-}$ samples. (A) Genes expressed at higher levels in WT neurons than NPCs. (B) Genes expressed at lower levels in WT neurons than NPCs. P-value were corrected by Benjamini-Hotchberg method.

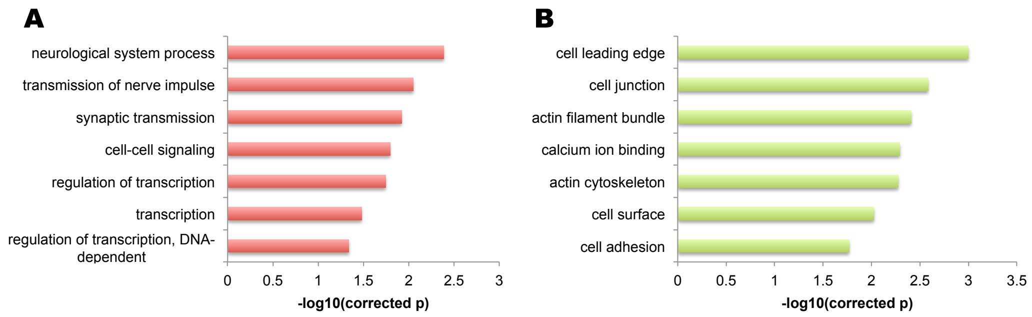

Figure S2: Expression of *CHD8* and brain volume associated DEGs in Brainspan. Expression values log2(FPKM+1) were scaled by row in heatmap. The numbers in parentheses below gene names represent Pearson's correlation coefficients (R) and p-values between *CHD8* and the corresponding gene. The order of genes was based on the correlation coefficients.

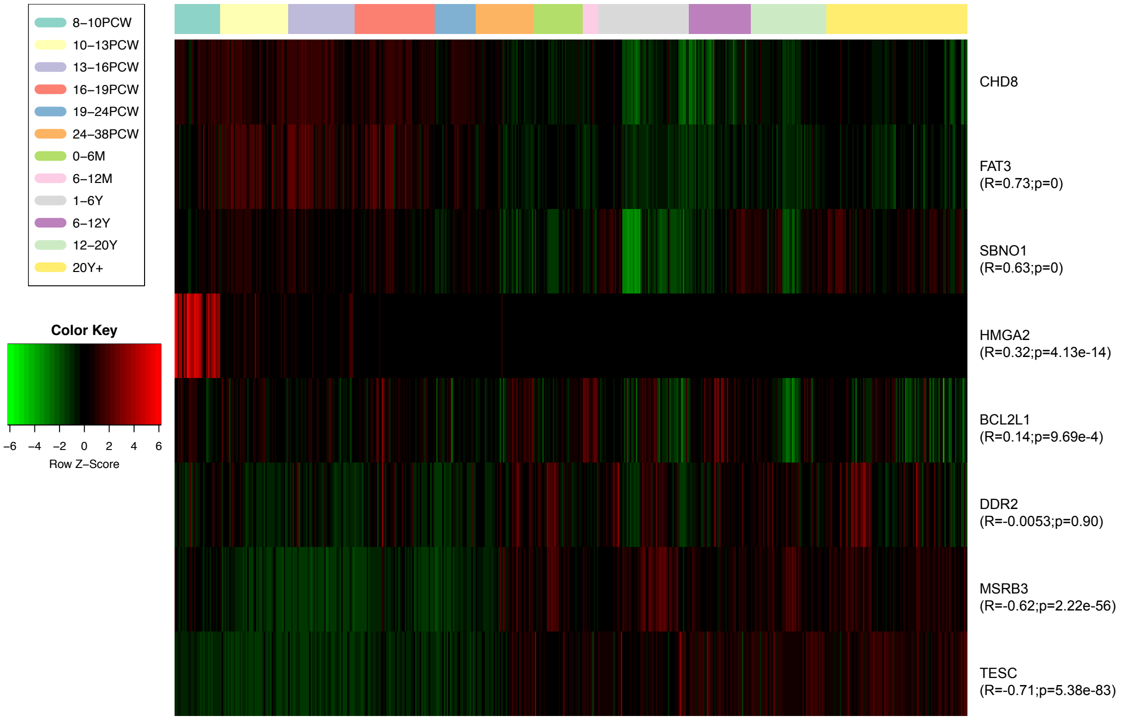

Figure S3: Comparison of the DEGs from current *CHD8*$^{+/-}$ analysis and previous *CHD8* knockdown studies. Genes were from NPC *CHD8*$^{+/-}$ (current data), NPC knockdown [1], NSC knockdown by two independent shRNAs (shC and shG) [2], SK-N-SH knockdown [3]. Also included DEGs from a previous transcriptomics profiling of organoids derived from ASD cases [4], with genes list from the TD11 and TD31 samples being combined. The red shading in each cell corresponds to the $-\log_{10}$P value for overrepresentation (Fisher's exact test, one-tailed), as shown in the color scale on the right (shown only if $p<0.05$). The first and the second number in each cell are the number of overlapping genes and the odd ratio of overlap to non-overlap, respectively. Numbers in the diagonal (grey cells) are the numbers of DEGs from previously studies that were also expressed in our samples.

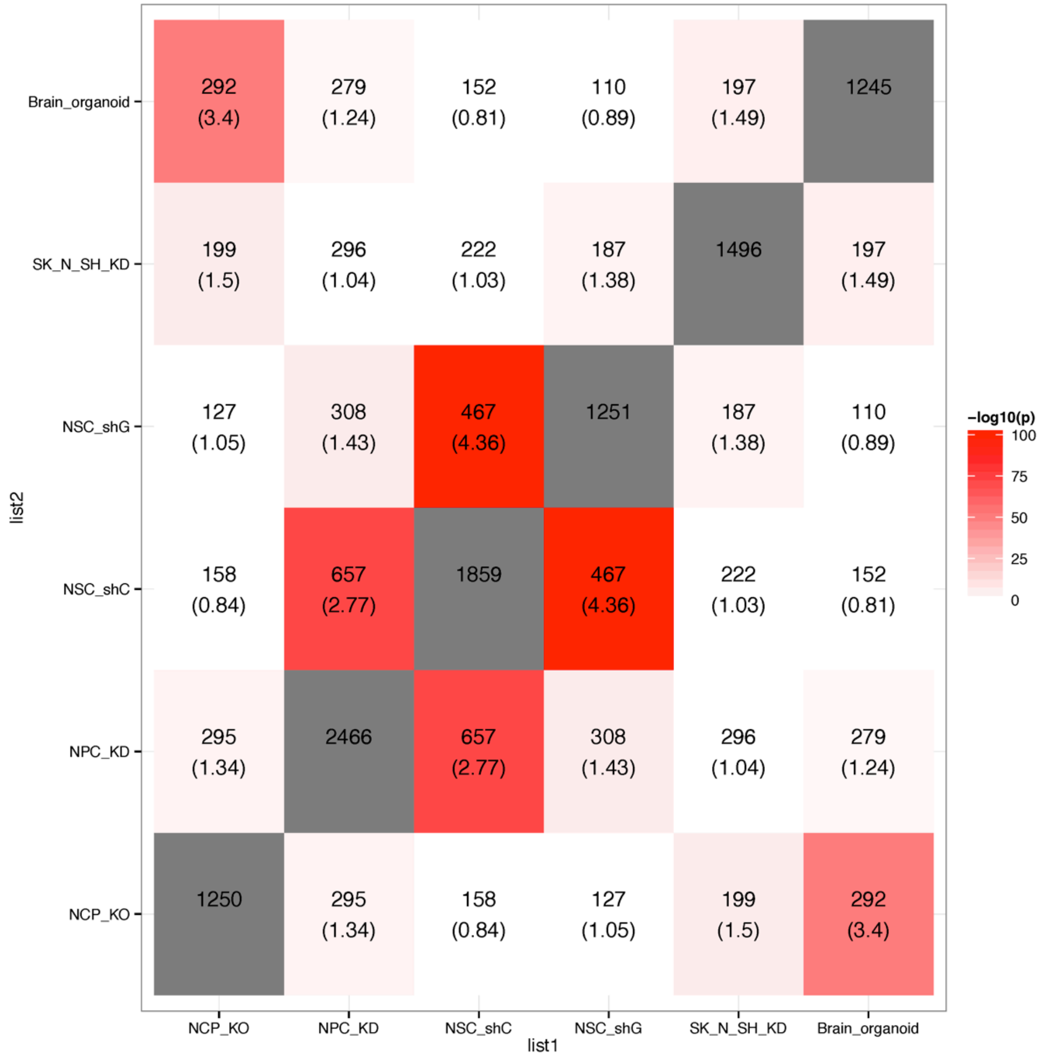

Table S1: Summary of RNA-seq quality.

Sample Name	Number of Pairs	Number of Mapped Pairs	exonic rate	intronic rate	intergenic rate	Median FPKM (genes)	Mean FPKM (genes)	Num of Genes (FPKM > 1)
control1_NPC	27,240,051	23,463,095	0.6088	0.34	0.0512	1.4672	67.8968	12,878
control2_NPC	24,248,402	21,275,566	0.7087	0.2567	0.0346	1.6287	110.7046	13,096
KO1_NPC	29,955,073	26,832,796	0.6494	0.3091	0.0415	1.8454	83.6047	13,294
KO2_NPC	24,531,882	21,941,336	0.6512	0.3092	0.0396	1.7143	95.6639	13,136
control1 _neuron	30,352,377	26,864,628	0.6307	0.3197	0.0496	1.6354	101.8777	13,158
control2_neuron	26,833,384	23,538,348	0.7356	0.221	0.0434	2.1256	107.5034	13,589
KO1_neuron	32,077,864	28,112,380	0.6372	0.3216	0.0412	2.6096	123.6631	14,062
KO2_neuron	28,613,352	24,164,270	0.7064	0.256	0.0376	2.5386	113.1035	14,030

References

1. Sugathan A, Biagioli M, Golzio C, Erdin S, Blumenthal I, Manavalan P, Ragavendran A, Brand H, Lucente D, Miles J, et al: **CHD8 regulates neurodevelopmental pathways associated with autism spectrum disorder in neural progenitors.** *Proc Natl Acad Sci U S A* 2014, **111:**E4468-4477.
2. Cotney J, Muhle RA, Sanders SJ, Liu L, Willsey AJ, Niu W, Liu W, Klei L, Lei J, Yin J, et al: **The autism-associated chromatin modifier CHD8 regulates other autism risk genes during human neurodevelopment.** *Nat Commun* 2015, **6:**6404.
3. Wilkinson B, Grepo N, Thompson BL, Kim J, Wang K, Evgrafov OV, Lu W, Knowles JA, Campbell DB: **The autism-associated gene chromodomain helicase DNA-binding protein 8 (CHD8) regulates noncoding RNAs and autism-related genes.** *Transl Psychiatry* 2015, **5:**e568.
4. Mariani J, Coppola G, Zhang P, Abyzov A, Provini L, Tomasini L, Amenduni M, Szekely A, Palejev D, Wilson M, et al: **FOXG1-Dependent Dysregulation of GABA/Glutamate Neuron Differentiation in Autism Spectrum Disorders.** *Cell* 2015, **162:**375-390.
