# Supplementary material for: CRISPR/Cas9-mediated heterozygous knockout of the autism gene CHD8 and characterization of its transcriptional networks in neurodevelopment
Source: Mol Autism. 2015 Oct 19;6:55. doi: 10.1186/s13229-015-0048-6 (PMC4612430; doi:10.1186/s13229-015-0048-6)
Supplement: Additional file 10: — Table S9–S10. Numbers and putative functionally disrupted coding variants in the WT iPSC line. (PDF 34 kb) [file 13229_2015_48_MOESM10_ESM.pdf]

Table S9: Number of variants of ips2 in ASD-risk genes.

type	No. of variants
frameshift INDEL	4
nonframeshift INDEL	3
nonsynonymous SNV	287
stopgain SNV	1
stoploss SNV	0
synonymous SNV	480

Table S10: Deleterious variants of ips2 in ASD-risk genes and their functional annotations by the ANNOVAR.

Chr	Position	Ref	Alt	Geno-type	Gene	Function	AAChange	Alt allele frequency (ESP6500)	Alt allele frequency (1KG)
8	100133706	T	G	1/1	VPS13B	stopgain	NM_181661:exon8:c.T1239G:p.Y413X	0.76	0.71
7	151945071	G	GT	0/1	KMT2C	frameshift insertion	NM_170606:exon14:c.2448_2448delinsAC	NA	NA
11	76954788	T	TA	0/1	GDPD4	frameshift insertion	NM_182833:exon12:c.1192_1192delinsTA	0.27	0.25
18	42456670	C	CTCTT	1/1	SETBP1	frameshift insertion	NM_001130110:exon4:c.681_681delinsCTCTT	0.53	0.49
22	19189003	A	AC	1/1	CLTCL1	frameshift insertion	NM_001835:exon23:c.3602_3602delinsGT,CLTCL1:NM_007098:exon23:c.3602_3602delinsGT	0.99	0.99
